# RNA-based therapeutic interventions for the management of Anderson-Fabry disease

**DOI:** 10.1016/j.omtn.2025.102679

**Published:** 2025-08-26

**Authors:** Stefano Cagnin

**Affiliations:** 1 Department of Biology, University of Padova, 35131 Padova, Italy; 2 CIR-Myo Myology Center, University of Padova, 35131 Padova, Italy

## Main text

**Figure 1:**
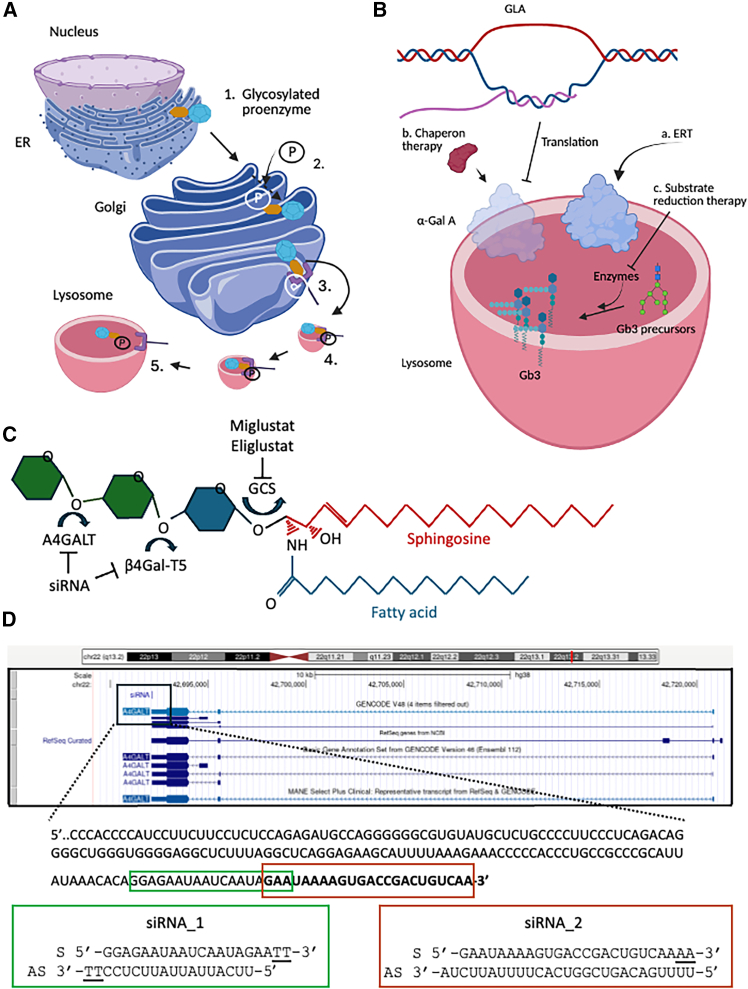
Therapeutic approaches for Fabry disease (A) Proenzymes possess an N-terminal sequence that guides their translocation into the lumen of the endoplasmic reticulum (ER), where the signal sequence is subsequently cleaved. Subsequently, the proenzyme undergoes N-linked glycosylation (1). Upon reaching the cis-Golgi apparatus, the mannose present on the lysosomal enzyme undergoes phosphorylation (2), resulting in the formation of mannose 6-phosphate (M6P), the recognition signal for lysosomal enzyme sorting. The M6P is subsequently recognized by M6P receptors in the trans-Golgi network (3), which directs the lysosomal enzymes toward the endo-lysosomal pathway. Lysosomal enzymes are subsequently packaged into clathrin-coated vesicles that bud from the trans-Golgi network (4) and reach the lysosome for fusion (5). (B) The GLA gene is transcribed to produce α-Gal A enzyme. In the event of mutations, the enzyme within lysosome does not operate or operates inefficiently inducing the accumulation of Gb3. Alternative approaches to counteract this phenomenon are the enzyme replacement therapy (a), the utilization of chaperons to stabilize partially functional enzymes (b), or to limit the synthesis of Gb3 by blocking enzymes processing precursors of Gb3 (c). (C) The ceramide composed by a sphingosine and fatty acid molecules is linked to a glucose through the glucosylceramide synthase (GCS) can be blocked by miglustat or eliglustat. Then the enzyme β-1,4-galactosyltransferase 5 (β4Gal-T5) adds a molecule of galactose to glucosylceramide producing a molecule of lactosylceramide. Finally, by the activity of lactosylceramide α-1,4-galactosyltransferase (A4GALT), Gb3 is produced. (D) A4GALT gene structure with transcript isoforms. Black enlarged box represents the mRNA sequence of the 3′-UTR where the two siRNAs bind. Green box represents siRNA 1 structure while red one represents siRNA 2. Underlined letters represent deoxyribonucleotides. S, sense; AS, antisense.

Recently, it has been demonstrated that the modulation of the expression of alpha 1,4-galactosyltransferase (A4GALT) serves as an alternative to mitigate the accumulation of globotriaosylceramide (Gb3) in endothelial cells (ECs) and podocytes that lack galactosidase alpha (GLA).^1^ GLA is a gene that encodes the homodimeric glycoprotein alpha-galactosidase A (α-Gal A). This enzyme is situated within lysosomes and catalyzes the hydrolysis of terminal α-galactosyl residues from glycolipids and glycoproteins. α-Gal A is synthesized as a preproenzyme, undergoes glycosylation, and is transported to lysosomes via the mannose-6-phosphate receptor pathway (Figure 1A). Under normal conditions, α-Gal A cleaves the α-1,4-galactosidic bond in Gb3, resulting in progressive degradation of Gb3. Mutations in the GLA gene cause Anderson-Fabry disease (FD), initially identified in 1898 by the two physicians who gave the name to the pathology. This pathology is an X-linked disease that predominantly affects males that have only one X chromosome, while females can be carriers or may exhibit milder symptoms due to the presence of a second, normal X chromosome. In females the disease’s penetrance is influenced by X chromosome inactivation or lyonization. Approximately 1:17,000 to 1:117,000 white males are affected by this condition.^2^

Patients accumulate Gb3 in various cells and tissues throughout the body, particularly in the blood vessels, kidneys, heart, skin, and nervous system. FD manifests into two different forms: classic and late onset. The classic form is less common and more severe. In this form, patients lack active α-Gal A enzymes, leading to an early pathology appearance during childhood or adolescence. Conversely, the late-onset form is characterized by the production of α-Gal A with a residual activity exceeding 1%. This allows the onset of symptoms after 30 years of age, but it also involves major organs such as the heart or kidneys.

This evidence underscores the paramount significance of newborn screenings.^3^ In fact, even if there are no definitive cures for FD, two Food and Drug Administration (FDA) and European Medicines Agency (EMA) approved alternatives are available to slow down the buildup of the fatty substances that primarily affect kidney, hearth, and blood vessels: enzyme replacement therapy (ERT) and chaperone therapy. From an ethical standpoint, it may be controversial to be aware of the possibility of developing a pathology for which there are no definitive treatments. However, an early diagnosis of later-onset forms may offer several advantages. The implementation of newborn screenings could prevent patients from remaining undiagnosed for years, enabling timely treatment and, consequently, improved outcomes,^4^ leading to different life decision, including lifestyle, financial, and reproductive choices.^5^ To mitigate the accumulation of fatty substances (Gb3) alleviating symptoms, slow disease progression, and enhance the quality of life, patients with FD may replace lacking enzyme (α-Gal A) (Figure 1B). ERT entails regular infusions of synthetic α-GAL A. Fabrazyme is a solution of recombinant agalsidase beta enzyme, a recombinant form of human α-galactosidase A produced using recombinant DNA technology from Chinese hamster ovary cell cultures. It is administered intravenously once every 2 weeks at a concentration of 1 mg per kg of body weight. To comprehend the administration process, it is crucial to note that Fabrazyme must be administered at the maximal velocity of 15 mg/h, resulting in an administration time exceeding 4 hours and 30 minutes for a male weighting 70 kg.

Moreover, since agalsidase beta (r-hαGAL) is a recombinant protein, the development of immunoglobulin G antibodies is expected in patients with little or no residual enzyme activity. ERT is a solution approved from FDA for several disorders (Table 1), but, in addition to previously discussed side effects, there are concerns regarding the elevated costs (usually over US$ 100,000/patient per year) and the difficulty to reach target tissues such as the central nervous system, bone, cartilage, cornea, and heart. The development of targeted enzyme delivery through enzyme modifications (e.g., based on receptor-mediated lysosomal enzyme delivery), nanosystems as nanoparticles, or the gene delivery based on adeno-associated viruses can be possible alternatives. Lipid nanoparticles (LNPs) were utilized to deliver mRNA encoding α-Gal A in mice and non-human primates, demonstrating its feasibility. This approach resulted in enhanced enzyme persistence in serum compared to ERT. α-Gal A was expressed in the liver, spleen, and kidneys within lysosomes. Notably, clearance of Gb3 was comparable to ERT.^6^

**Table 1:** Approved enzyme replacement therapies

Disease	Deficient enzyme	Inheritance	Brand name and approval year
Mucopolysaccharidosis I (Hurler syn.)	α-L-iduronidase	autosomal	Aldurazyme/2003 FDA, EMA
Mucopolysaccharidosis II (Hunter syn.)	iduronate sulfatase	X-linked	Elaprase/2006 FDA; 2007 EMA
Mucopolysaccharidosis IV A (Morquio A syn.)	N-acetylgalactosamine 6-sulfatase	autosomal	Vimzim/2014 FDA; EMA
Mucopolysaccharidosis VI (Marateaux-Lamy syn.)	N-acetylgalactosamine 4-sulfatase	autosomal	Naglazyme/2005 FDA; 2006 EMA
Acid sphingomyelinase deficiency	acid sphingomyelinase	autosomal	Xenpozyme/2022 FDA; EMA
Alpha-mannosidosis	α-mannosidase	autosomal	Lamzede/2023 FDA; 2018 EMA
Fabry disease	α-galactosidase	X-linked	Fabrazyme/2001 EMA; 2003 FDA and 2021 FDA for pediatric patients older than 2 years
			Replagal/2003 EMA
			Elfabrio/2023 FDA; EMA
Pompe disease	α-glucosidase	autosomal	Myozyme/2006 FDA, EMA
			Nexviadyme/2022 EMA
			Pombiliti (used in combination with Opfolda to treat late onset Pompe disease/2023 FDA; EMA
			Lumizyme/2010 FDA
Gaucher disease	β-glucocerebrosidase	autosomal	Ceredase/1991 FDA
			Cerezyme/1994 FDA; 1997 EMA
			Zavesca/2003 FDA; 2002 EMA
			Cerdelga/2014 FDA; 2015 EMA
			VPRIV/2010 FDA, EMA
			Elelyso/2012 FDA
Lysosomal acid lipase deficiency	lysosomal acid lipase	autosomal	Kanuma/2015 FDA, EMA

A second alternative for the treatment of patients with FD is Galafold. This oral medication functions by stabilizing the mutated enzyme, enabling it to reach the lysosomes and carrying out its function (Figure 1B). Therefore, this drug can be used in specific cases when the GLA mutations permit to produce α-Gal A with a residual enzymatic activity. Thus, if this therapy is innovative because patients can take a pill instead of a transfusion as for the ERT, not all patients can benefit from Galafold.

A third alternative for the treatment of FD is to limit the synthesis of Gb3 (Figure 1B): the substrate reduction therapy. Gb3 is a glycosphingolipid formed by a lipid skeleton (sphingosine and a fatty acid linked through an ammine bond that makes them a ceramide molecule) connected to an oligosaccharide (Figure 1C). The ceramide part of the molecule is synthesized in the cytosolic part of endoplasmic reticulum membranes by ceramide synthases (CerS1-6) while the synthesis of oligosaccharide takes place in the Golgi apparatus. Firstly, a glucose molecule is linked to the ceramide by the enzyme glucosylceramide synthase (GCS) producing a glucosylceramide (GlcCer). Interestingly, this step can be modulated by using different drugs: miglustat (Zavesca) and eliglustat (Figure 1C). They are approved to treat Gaucher disease type 1. Glucosylceramide is then modified thorough the enzyme β-1,4-galactosyltransferase 5 (β4Gal-T5) that adds a molecule of galactose to form lactosylceramide (LacCer). Finally, Gb3 (galactose α-1,4 galactose β-1,4 glucose 1,1′ Cer) is produced by adding a molecule of α-1,4 galactose to LacCer. This last step is catalyzed by the enzyme lactosylceramide α-1,4-galactosyltransferase (Gb3 synthase synthesized by the gene A4GALT) (Figure 1C). This is the enzyme that was chosen from Shin and colleagues^1^ to limit the accumulation of Gb3 in cells used as models for FD. Three fundamental considerations must be addressed when using RNA in the treatment of lysosomal storage disorders: (1) the precise selection of the gene to be targeted, (2) the appropriate method for RNA delivering, and (3) the necessity of ongoing therapy administration.

In response to the first criticism, Shin and colleagues chose to focus on Gb3 synthase rather than targeting the GCS transcript, as was done by Diaz-Font et al.^7^ to demonstrate the therapeutic efficacy for Gaucher disease (Figure 1C). Indeed, as Shin and colleagues have elucidated, their approach does not induce any adverse effects on the synthesis of essential glycosphingolipids, such as GlcCer and LacCer. The second criticism regards the RNA delivery. Indeed, RNA must reach the target tissue, and several approaches have been explored and used in approved RNA-based therapies.^8^ Shin and colleagues evaluated the possibility of using polyhistidine (p-His) in the formulation of LNPs. P-His facilitates endosomal escape of LNPs, while p-His interacts with RNAs through ionic forces limiting their ability to be encapsulated in LNPs. Therefore, a specific p-His/small interfering RNA (siRNA) weight ratio (0.2) was selected for histidine-LNP formulation to encapsulate both p-His and siRNA into LNPs simultaneously.^1^ siRNA-histidine-LNPs targeting A4GALT exhibited no impact on α-GAL A activity indicating the absence of regulatory loops. This strategy facilitates molecular recovery, particularly in ECs derived from induced pluripotent stem cells knockout for GLA gene, compared to podocyte-derived cells (PCs).^1^ This disparity was attributed to observed variations in transfection efficacy between ECs and PCs, underscoring the significance of the delivery method and its specificity for cells and tissues.

The third criticism was not directly addressed in the work of Shin and colleagues, but the possibility to augment siRNA delivery efficacy through p-His-LNPs may prolong its efficacy. Other methods that may be used for the purpose are based on the cell transfection with DNA vectors bearing short hairpin RNAs, but they present the same problems associated with the delivery of genes. Alternatives to p-His-LNPs include PEGylated lipids, which enhance macrophage evasion, thereby extending circulation duration or stable nucleic acid lipid particles (SNALPs). SNALPs are novel lipid bilayer containing a mixture of cationic and fusogenic lipids coated with diffusible polyethylene glycol that facilitate relatively low dosing of siRNAs maintaining long-term action.^9^

In addition to the importance of delivery efficacy, the stability, structure, and length of RNA were discussed by Shin and colleagues. They demonstrated that distinct siRNA-binding sites and varying siRNA lengths influence their efficacy in downmodulating target gene.^10^ Interestingly, for optimal downmodulation of Gb3 synthase, siRNAs must bind 3′UTR of the gene and must be modified adding two deoxyribonucleotides at their 3′ end (Figure 1D).^10^

In conclusion, Shin and colleagues demonstrated the feasibility of siRNA therapy by utilizing *in vitro* platforms (hiPSC [human induced pluripotent stem cells]-derived ECs and PCs) that exhibit FD phenotypes. These platforms demonstrated enhanced RNA cellular entry and biodistribution facilitated by modified LNPs, leading to membrane disruption and a proton sponge effect mediated by p-His. Although the method should be tested for long-term therapeutic viability and safety using *in vivo* models, it may also be considered, as in the case of all RNA-based treatments, not as an alternative to previously discussed therapeutic options but as an adjuvant therapy to use together with other approaches, such as the chaperone therapy.

## Acknowledgments

We acknowledge the support provided by the 10.13039/100000002National Institutes of Health (NIH) under grant no. 1R21NS139044-01 and the Research Projects of Relevant National Interest 2022NBFJNT.

## Author contributions

S.C. wrote the manuscript.

## Declaration of interests

The author declares no competing interests.
